# Microplasma-synthesized ultra-small NiO nanocrystals, a ubiquitous hole transport material†

**DOI:** 10.1039/c9na00299e

**Published:** 2019-10-22

**Authors:** Supriya Chakrabarti, Darragh Carolan, Bruno Alessi, Paul Maguire, Vladimir Svrcek, Davide Mariotti

**Affiliations:** Nanotechnology & Integrated Bio-Engineering Centre (NIBEC), Ulster University Jordanstown, Newtownabbey Co. Antrim BT37 0QB UK; Centre for Carbon Materials, International Advanced Research Centre for Powder Metallurgy and New Materials (ARCI) Balapur P.O. Hyderabad 500005 India supriya.c@arci.res.in supriyac79@gmail.com; National Institute of Advanced Industrial Science and Technology (AIST), Department of Energy and Environment, Research Center of Photovoltaics, Advanced Processing Team Central 2, Umezono 1-1-1 Tsukuba Ibaraki 305-8568 Japan

## Abstract

We report on a one-step hybrid atmospheric pressure plasma-liquid synthesis of ultra-small NiO nanocrystals (2 nm mean diameter), which exhibit strong quantum confinement. We show the versatility of the synthesis process and present the superior material characteristics of the nanocrystals (NCs). The band diagram of the NiO NCs, obtained experimentally, highlights ideal features for their implementation as a hole transport layer in a wide range of photovoltaic (PV) device architectures. As a proof of concept, we demonstrate the NiO NCs as a hole transport layer for three different PV device test architectures, which incorporate silicon quantum dots (Si-QDs), nitrogen-doped carbon quantum dots (N-CQDs) and perovskite as absorber layers. Our results clearly show ideal band alignment which could lead to improved carrier extraction into the metal contacts for all three solar cells. In addition, in the case of perovskite solar cells, the NiO NC hole transport layer acted as a protective layer preventing the degradation of halide perovskites from ambient moisture with a stable performance for >70 days. Our results also show unique characteristics that are highly suitable for future developments in all-inorganic 3^rd^ generation solar cells (*e.g.* based on quantum dots) where quantum confinement can be used effectively to tune the band diagram to fit the energy level alignment requirements of different solar cell architectures.

## Abstract 2

We report on a one-step hybrid atmospheric pressure plasma-liquid synthesis of ultra-small NiO nanocrystals (2 nm mean diameter), which exhibit strong quantum confinement and excellent compatibility as hole transport layer for various solar absorber layers.

## Graphical abstract

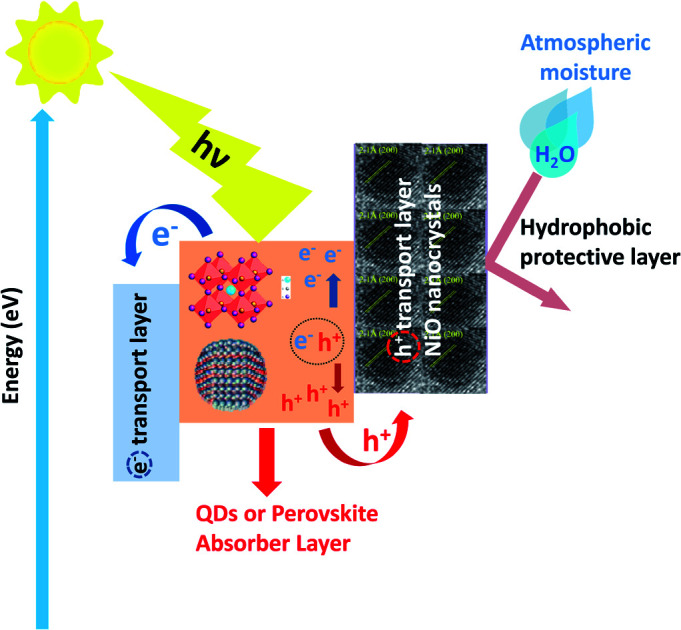

## 1. Introduction

Atmospheric pressure hybrid plasma-liquid synthesis of inorganic nanocrystals is an emerging technology and a powerful alternative to other chemical and physical synthesis methods.^1–3^ This synthesis approach is a low-cost one-step solution for producing stable nanocrystals with a high degree of reproducibility. Materials with high purity can be obtained with this process and additional complex purification steps can be avoided. Following the production of nanomaterials in the form of colloids, spray coating can be employed for deposition on a large surface area as required for various applications. The spray deposition of thin films from nanocrystal colloidal solutions is developing as a prevailing alternative to conventional methods of film deposition, which rely on high-vacuum and high temperature processing of bulk semiconductors. In addition to the expected cost saving, the incorporation of spray processed NC films into device architectures offers simplicity, flexibility and an additional degree of freedom for large area multilayer device fabrication. Metal oxides are becoming ubiquitous as charge transport layers for all various types of next generation solar cells like perovskite, organic and quantum dot based solar cells. Metal oxides play key roles in ensuring selective carrier extraction, preserving a high open-circuit voltage as well as acting as a protective layer, *e.g.* from environmental moisture. Organic transport layers have been often used in various device architectures;^4–6^ however, metal oxides have demonstrated much better performance with high carrier mobility and better stability.^7–10^

For instance, among various organic transport layers Spiro-MeOTAD (*N*^2^,*N*^2^,*N*^2′^,*N*^2′^,*N*^7^,*N*^7^,*N*^7′^,*N*^7′^-octakis(4-methoxyphenyl)-9,9′-spirobi[9*H*-fluorene]-2,2′,7,7′-tetramine) and PEDOT:PSS (poly(3,4-ethylenedioxythiophene)-polystyrene sulfonate) are two of the most widely used hole transport materials (HTMs), *e.g.* due to their favourable band alignment with perovskite absorbers. The organic HTM Spiro-MeOTAD requires a lengthy and low yielding synthetic process and costly sublimation steps for purification, which makes it highly expensive.^11–13^ In the pristine form, it exhibits low conductivity and low hole mobility and thus requires additives and chemical p-dopants. Spiro-MeOTAD, when heated (80 °C to 120 °C), it forms large voids that lead to the degradation of the device. PEDOT:PSS on the other hand is known to degrade the interface with the absorber layer in the device due to the acidic nature of PSS. This layer also easily absorbs water from the environment which finally damages the perovskite absorber layer. Therefore, the development of stable and cost-effective HTMs is imperative where inorganic oxides can play a key role.

In this context, p-type nickel oxide (NiO) possesses suitable features as an HTM^14,15^ for PV devices. NiO has a large bandgap and deep valence band which provides energetically favourable band alignment with the photo-absorber layer. As a hole transport layer, due to its hydrophobic^16^ nature, it can also provide good environmental stability.^17^

The use of NiO as a transport layer in various morphologies has been reported for different types of solar cells, including organic- and perovskite-based devices.^18–21^ For instance, Jiang *et al.*^22^ reported NiO_*x*_ nanoparticles as a highly efficient hole transport layer at room temperature for high performance organic optoelectronic devices. Also there are reports showing that non-stoichiometric NiO_*x*_ nanoparticles can form an efficient hole transport layer at room temperature for high performance solar cells with different polymer or perovskite active layers.^23–28^ Zhang *et al.*^23^ demonstrated a surface-nanostructured NiO_*x*_ film formed by a room-temperature solution process for achieving high performance flexible perovskite solar cells with good stability and reproducibility. Irwin *et al.*^24^ showed that a thin (5–80 nm) layer of NiO as an anode layer can minimize the interfacial power losses and enhance the efficiency of polymer bulk-heterojunction solar cells. Some recent studies indicated that NiO combined with some other materials like graphene oxide (GO)^29^ and PbI_2_ ^30^ can further improve the properties of the hole transport layer including the film quality, passivation of defects, suppression of dark current and enhancement of photocurrent. The nanocomposite structures work as an effective hole extraction layer, which provides a large electron injection barrier and favourable hole extraction as well as passivating the surface of the perovskite for achieving a stable high efficiency solar cell device.

These results indicate the superiority and potential of using NiO as a hole transport layer in photovoltaic devices compared to that of organic based HTMs. There have been several approaches to prepare NiO films including sol–gel processes, electro-deposition, sputtering and pulsed laser deposition, which could be adopted for the fabrication of organic PV (OPV) devices and dye-sensitized solar cells (DSSC).^15,16,31^ However, all these processes involve complex purification steps.

Here, we report the synthesis of high purity, stable and quantum confined NiO nanocrystals (NCs) with a narrow size distribution using a hybrid plasma-liquid one-step synthesis technique. We have performed in-depth material characterization of the NiO NCs as well as measurements to determine important band energy parameters. The absolute position of the band-edges, bandgap and Fermi level of the NiO NCs is affected by the quantum confinement regime, which can be beneficial for application in PV devices. These parameters could all be tuned precisely depending on the application and device structure (different for different absorbers) through quantum confinement and by simply optimizing the particle sizes. The tunability of the highest occupied molecular orbital and lowest unoccupied molecular orbital (HOMO/LUMO) levels is possible through doping for organic hole transport layers like Spiro-MeOTAD and PEDOT:PSS; however the doping processes are complex and expensive.^32–34^ We have therefore integrated NiO NCs layers into three different next generation device architectures. A colloidal sol with NiO NCs was first spray coated onto a perovskite layer to form a uniform HTM. We fabricated a full perovskite device structure under open ambient conditions and demonstrated improved stability. The perovskite solar cell with the NiO NC hole transport layer showed high stability over 70 days compared to the perovskite cell without the NiO NC layer; an increase in the power conversion efficiency has also been observed. Silicon quantum dot (Si-QD) and nitrogen-doped carbon quantum dot (N-CQD) based solar cells with NiO NC hole transport layers were also studied here and the result indicate their rectifying nature with a high *V*_oc_. These results show great opportunities for both the integration of the synthesis approach into device fabrication steps as well as for quantum confined NCs for optimized transport layers.

## 2. Results and discussion

### 2.1. NiO NC synthesis, characterization and properties

**Fig. 1:**
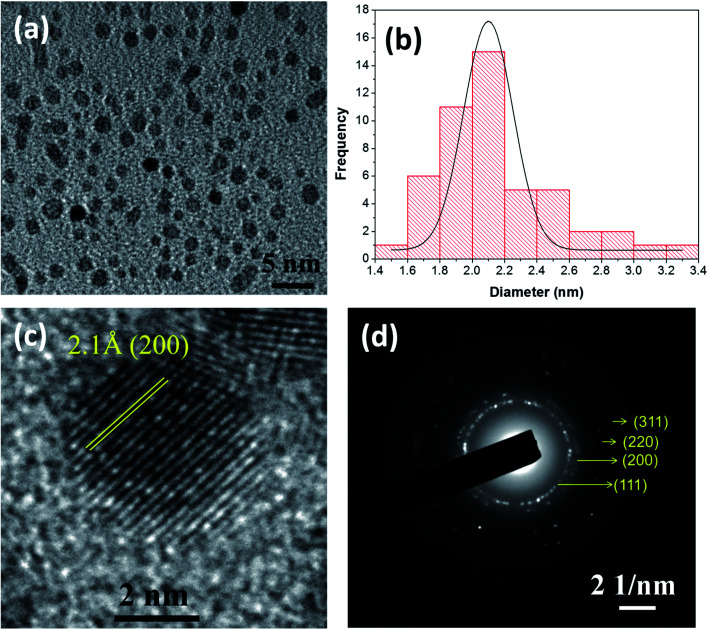
(a) TEM micrograph of as-grown NiO nanocrystals prepared by atmospheric pressure hybrid plasma-liquid synthesis. (b) Histogram of NiO nanocrystals obtained from the TEM image (a). (c) HRTEM image of NiO nanocrystals showing the lattice spacing. (d) SAED pattern of the as-grown NiO nanocrystals.

NiO NCs were synthesized using an atmospheric pressure direct current microplasma interacting with the liquid phase^35^ (see the ESI† for the detailed experimental procedure). The schematic diagram and digital photograph of the atmospheric pressure hybrid plasma-liquid synthesis unit are shown in Fig. S1a and b† respectively. Transmission electron microscopy (TEM) was used to characterize the particle size distribution and crystallinity. Fig. 1a presents the TEM micrograph of the synthesized NiO NCs which shows a narrow size distribution, see Fig. 1b. The histogram (Fig. 1b) corresponding to the particle size distribution and fitting the histogram to a Gaussian function yielded a mean diameter of 2.1 nm, with a standard deviation of 1 nm. High resolution TEM images (Fig. 1c) and the selected area electron diffraction (SAED) pattern (Fig. 1d) have been used to study the crystal structure of the NCs, which indicated a face-cantered cubic structure of NiO.^36^

**Fig. 2:**
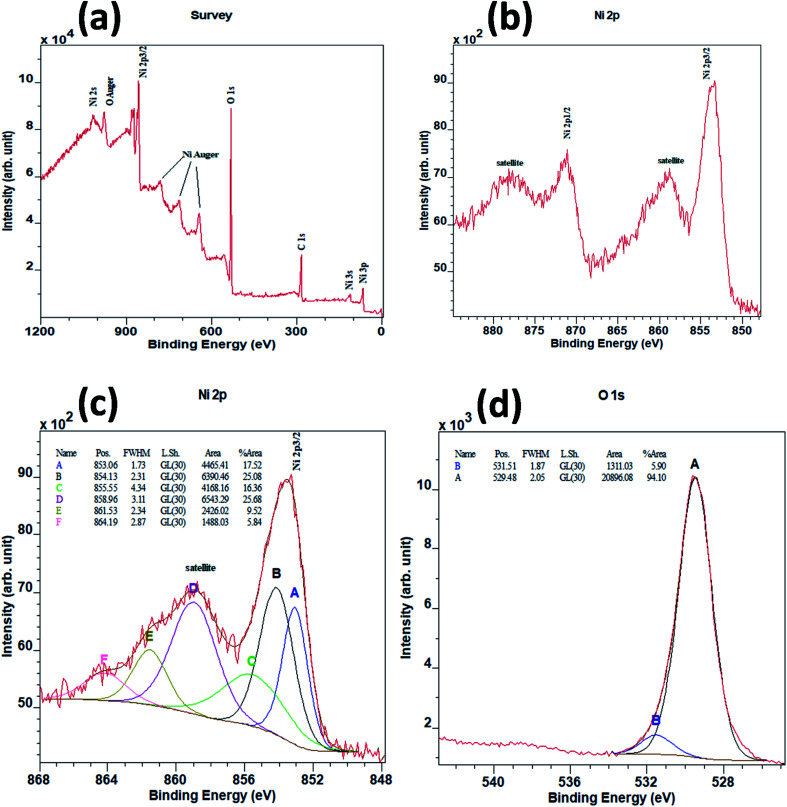
(a) High-resolution XPS survey spectrum of the NiO NC film. (b) 2P spectrum of NiO NCs. (c) XPS spectrum of Ni 2p_3/2_ peaks of NiO NCs and (d) XPS spectrum of O 1s peaks of NiO NCs.

To understand the chemical composition and electronic structure of the NiO NCs, we have carried out high resolution X-ray photoelectron spectroscopy (XPS) analysis. Fig. 2a shows the XPS survey spectrum of the NiO NC film, which consists of signals from Ni, O, and C. The shape and the position of Ni and O Auger signals are well matched with earlier reported data of NiO.^37,38^ The high resolution Ni 2p region XPS spectrum (Fig. 2b) shows four distinct features, the Ni 2p_3/2_ main peak and its satellite at ∼854 and ∼859 eV, and the Ni 2p_1/2_ main peak and its satellite at ∼871 and ∼878 eV, respectively. As a first-row transition metal oxide, NiO has a complex 2p spectrum because of peak asymmetries, complex multiplet splitting, a shake-up process, final state effects, and uncertain overlapping binding energies.^39^ Photoionization creates core electron vacancies in NiO and strong coupling occurs between the unpaired electrons of the core and outer shell. As a result, multiplet splitting occurs and forms a number of final states, which result in a broad XPS Ni 2p spectrum (Fig. 2b). To understand the complex electronic states of NiO, the broad 2p spectrum needs to be de-convoluted into multiple peaks. Fig. 2c shows the XPS spectrum of Ni 2p_3/2_, which has been de-convolute into six peaks and denoted by A to F in the figure. These peak positions are in good agreement with previously reported NiO films synthesized by various techniques.^38,40–44^

The lowest binding energy peak at 853.1 eV (peak A) can be attributed to the cd^9^L state of NiO.^45,46^ The main peak centred at 854.1 eV (peak B) indicates the chemical state of Ni^2+^ in the standard NiO_6_ octahedral bonding configurations of the cubic rock-salt NiO structure.^47,48^ The peak centred at 855.5 eV (peak C) has been generally attributed to the vacancy-induced Ni^3+^ ion^47,49^ or nickel hydroxides and oxyhydroxides.^50–54^ However, more recently, peak C has also been interpreted as the combination of more than a single state due to non-local screening processes and with a partial contribution from the surface states of NiO.^55,56^ This more recent interpretation is now the accepted origin for the appearance of peak C.^55,56^ The peaks at around 859 eV (peak D) and 862 eV (peak E) are unanimously attributed to the cd^10^L^2^ and cd^8^ states of NiO respectively.^45,46^ The highest binding energy peak centred at 864 eV (peak F) in the 2p envelop is due to the shake-up transition^47^ in the NiO structure.

Fig. 2d shows the XPS spectrum for O 1s. Two peaks can be de-convoluted from the spectrum centered at 529.5 eV and 531.5 eV respectively. The peak centred at 529.5 eV can be assigned to Ni–O octahedral bonding in NiO^47^ and the peak centred at 531.5 eV is likely due to the presence of nickel hydroxides or to surface adsorbed hydroxyl groups.^53,54,57^

**Fig. 3:**
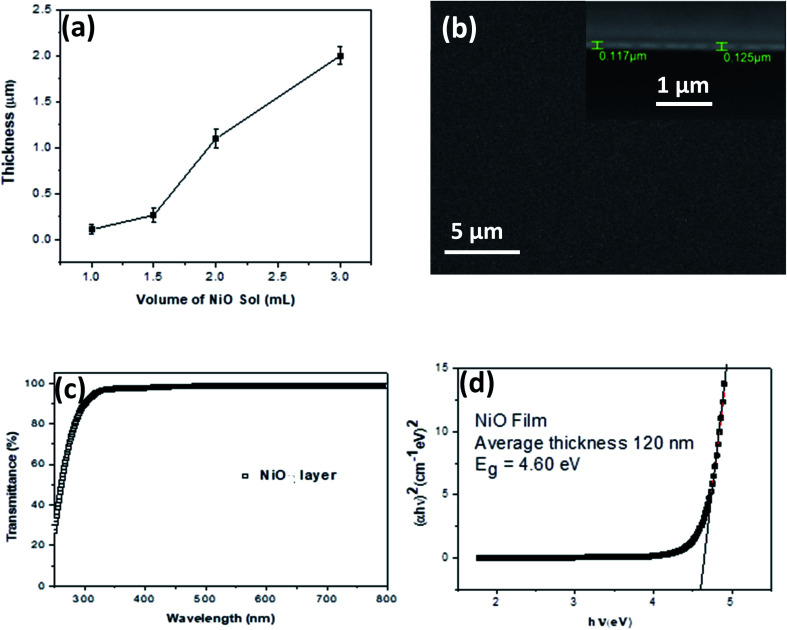
(a) The plot of NiO NC film thickness *vs.* spray volume of the NiO NC sol. The standard deviation indicates the variation in the NiO film thickness due to spray coating. (b) SEM image of the top surface of the NiO NC film deposited onto a Si substrate (inset: cross sectional SEM image of the NiO NC thin film showing the thickness of the film). (c) Optical transmittance spectrum of the NiO NC (∼120 nm) film on a quartz substrate and (d) Tauc plot of the NiO NC film revealing the bandgap of the material.

Continuous thin films of NiO NCs with various thicknesses have been deposited onto indium-doped tin oxide (ITO) coated glass substrates using spray coating of a NiO NC-sol *via* a nitrogen gas flow at 1 bar. A schematic diagram of the spray deposition system for NiO layer deposition is shown in Fig. S2a.† The thickness of the film can be adjusted by controlling the volume of the sol to be sprayed and it can be varied from 100 nm to 2000 nm (Fig. 3a). When 1 mL of NiO–ethanol sol was spray coated onto the substrate, the NiO NC film exhibits a continuous and smooth top surface which is evident from the scanning electron microscope (SEM) image shown in Fig. 3b. The corresponding cross-sectional SEM image of the NP film is shown in the inset of Fig. 3b, which reveals an average film thickness of 120 nm when spray coated with 1 mL of NiO sol. For understanding the crystallinity of the NiO NC film deposited using the spray coating technique, we have carried out an X-ray diffraction (XRD) study of the films. The XRD spectrum of the NiO NC film of thickness ∼2000 nm is shown in Fig. S2b,† which indicates two distinct peaks, (111) and (200) of cubic NiO.^36^

Depending on the device architecture, metal oxide transport layers are preferred to be highly transparent to minimize the optical losses across the visible (Vis) and near infrared (NIR) regions for photovoltaic devices. The optical transmittance spectrum of the 120 nm thick NiO NC film in the ultraviolet-Vis-NIR region is shown in Fig. 3c. A higher transparency (>90%) above the 400 nm wavelength was observed for NiO NC films, which is highly desirable for photovoltaic devices, as it generally leads to the enhanced light-harvesting capability of the absorber layer. The optical bandgap (*E*_g_) of the NiO NC film is determined by plotting (*αhν*)^1/*m*^*vs. hν* where *α* is the absorption coefficient. The value for *m* indicates the nature of the transition, *i.e.* 1/2, 2, 3/2 or 3 for allowed direct, allowed indirect, forbidden direct and forbidden indirect transitions, respectively.^58^ Assuming allowed direct transitions (*m* = 1/2) in our case, we have been able to estimate the bandgap (*E*_g_ = 4.60 eV) using a Tauc plot^59^ (Fig. 3d). A similar range of bandgap values have been previously reported for NiO nanocrystals.^60–62^ A clear blue shift in the bandgap of the NiO NCs compared to that of the bulk (3.7–4.0 eV)^63^ has been observed indicating quantum confinement of our NiO NCs. No linear relationship between (*αhν*)^1/*m*^ and *hν* was found for *m* = 2, 3/2 and 3 suggesting that the NiO NC films in this study possessed a direct allowed band gap.^64–66^

**Fig. 4:**
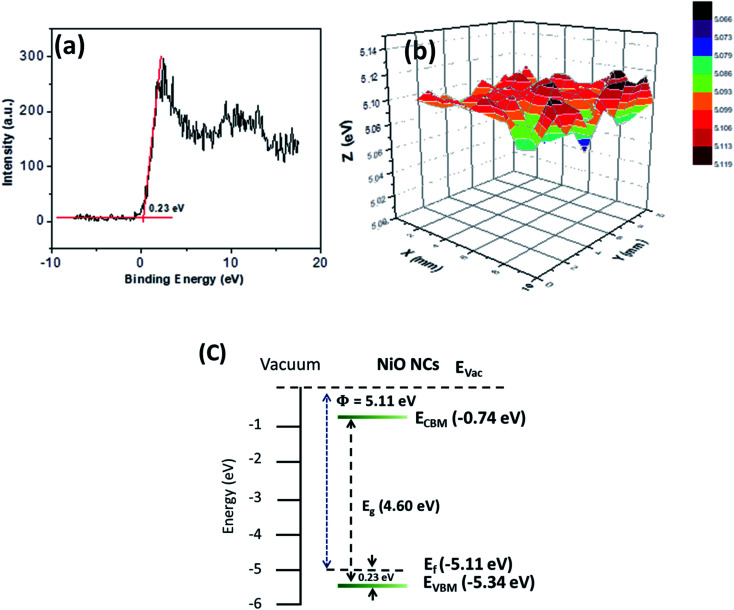
(a) Valence band maxima spectrum of the NiO NC film obtained from XPS spectroscopy. (b) Profile plot of the Fermi level over a certain area of the NiO NC film measured by using a Kelvin probe in scanning mode and (c) energy band diagram of NiO NCs devised from optical absorbance, XPS and Kelvin Probe measurements.

The energy band structure of the NiO NC film was further investigated using XPS and Kelvin probe measurements. The valence band maximum (VBM) of the NiO NC films was determined by linearly extrapolating the low binding energy edge of the valence band intersecting the background in the XPS spectra. The linear extrapolation of the valence band spectrum leading edges as shown in Fig. 4a gives the difference between the Fermi level and the valence band maximum for NiO NCs. This was found to be 0.23 eV which confirms the p-type nature of the material. Kelvin probe measurements in the scanning mode were used to study the work function (*Φ*) of the NiO NC film (Fig. 4b), which is related directly to the Fermi level; this was measured to be 5.11 eV. The profile plot of the Fermi level over a certain area indicates consistent electrical properties of the NiO NC film with a small variation in the value. The resulting energy band diagram of the NiO NC layer is shown in Fig. 4c. This band diagram shows the valence band maximum, conduction band minimum (determined by adding the bandgap to the valence band maximum), bandgap and Fermi level of the NiO NC layer with a thickness of 120 nm. All these values were directly obtained from our experimental measurements and the details are given above.

### 2.2 Solar cell architectures

The measured Fermi level and valence band maximum of the NiO NC layer align well with the energy level of the valence band (VB) of various absorber materials in solar cells such as Si quantum dots or C dots or CH_3_NH_3_PbI_3_ perovskites. Thus, the NiO NC layer with the optimised thickness can be an efficient hole transport layer (HTL) for charge transport with little energy loss. We have therefore integrated the NiO NCs as a hole transport layer into quantum dot (QD) based all-inorganic 3^rd^ generation solar cells. The energy levels of QDs are adjustable by varying their size, which finally defines the bandgap of the material. As a result, the bandgap values and HOMO/LUMO of the material can be tuned without changing the original material. These excellent tunable properties and low cost synthesis process make QDs desirable for future generation solar cells.

#### 2.2.1 QD based solar cell architectures

**Fig. 5:**
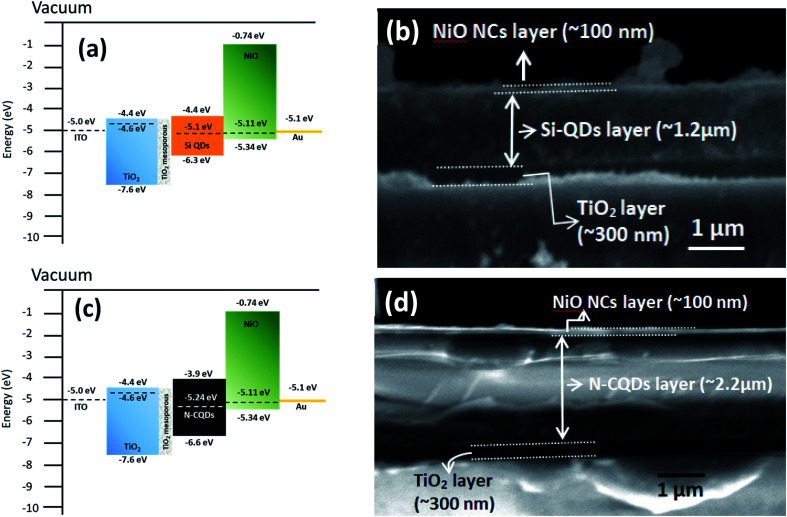
(a) Energy band alignment of the all-inorganic Si-QD based device structure consisting of glass/ITO/TiO_2_ compact layer/TiO_2_ mesoporous layer/Si-QDs/NiO/Au. (b) Cross sectional image of the device (glass/ITO/TiO_2_ compact layer/TiO_2_ mesoporous layer/Si-QDs/NiO) without the metal contact (Au) showing the thicknesses of TiO_2_, Si-QD and NiO layers. Some flakes of metal oxides are visible in the image which occurred during the cutting of the device for cross sectional SEM imaging. (c) Energy band alignment of the all-inorganic N-CQD based device structure consisting of glass/ITO/TiO_2_ compact layer/TiO_2_ mesoporous layer/N-CQDs/NiO/Au and (d) cross sectional image of the device (glass/ITO/TiO_2_ compact layer/TiO_2_ mesoporous layer/N-CQDs/NiO) without the metal contact (Au) showing the thicknesses of TiO_2_, N-CQD and NiO layers.

The band diagram of NiO NCs indicates that it can fit well as an HTL for Si^3,67,68^ and C^69^ QD based solar cells. The complete device structure is shown in Fig. S4a,† where the TiO_2_ layer works as an electron transport material, the Si-QD thin film works as an absorber and the NiO NC layer works as a hole transport material. The energy band diagram and band alignment for this device structure are shown in Fig. 5a. The cross-sectional SEM image of the layered device structure just before the metal contact is shown in Fig. 5b. The image clearly reveals the transport layers TiO_2_ and NiO NCs as well as the absorber layer Si-QDs in the device architecture. Fig. S4b† shows the current density–voltage (*JV*) plot for the Si-QD devices with the NiO NC hole transport layer, which indicates a FF of 35% and a *V*_oc_ of 0.64 V. The reasonably high open-circuit voltage is a result of the band alignment between the Si-QD and NiO NC layer. The initial result for the Si-QD based device is promising demonstrating the rectifying nature with high *V*_oc_. It is clearly evident from this result that the NiO NC HTL is effectively transporting the carriers to the metal contact.

In order to test the general applicability of NiO NCs for 3^rd^ generation QD based solar cells, devices with N-CQDs^69^ as an absorber were also fabricated. Fig. S4c† shows the schematic diagram of the complete device structure. The energy band diagram and band alignment of each layer are shown in Fig. 5c. The cross-sectional SEM image (Fig. 5d) shows the thicknesses of the NiO NC, N-CQD and TiO_2_ layers. The *JV* characteristics of the device are shown in Fig. S4d.† The device shows good rectification with a high *V*_oc_ (0.93 volt) and a FF of ∼40%. This performance can be attributed to efficient alignment of the Fermi levels of N-CQDs with the NiO NC and TiO_2_ carrier transport layers (Fig. 5c). As a proof of concept, we have shown here that NiO NCs worked well as an HTL for QD based solar cell devices. In this preliminary study the efficiencies are weak, especially for Si QDs. The efficiency for solar cells with C QDs is better due to reduced energy losses and a better band alignment. Further optimization of the device structure and layer thicknesses of Si-QD, N-CQD and carrier transport layers is needed to achieve high photocurrent and the work is in progress. However, these initial results show the general applicability of the NiO NC film fabrication process and device integration for a range of diverse architectures, demonstrating the feasibility of the fabrication processes based on atmospheric pressure plasmas.

#### 2.2.2 Perovskite based solar cell architecture

Furthermore, we have tested the performance of the NiO NC HTL in perovskite-based solar cells. Fig. S5a† shows the device structure of the complete metal oxide-perovskite solar cell. The details of the fabrication process of the layered device have been described in the Experimental section (see the ESI† for the detailed procedure). The device architecture consists of glass/ITO/TiO_2_/perovskite/NiO NCs/Au, in which TiO_2_ and NiO NC layers are working as the electron and hole transport layers, respectively, and the perovskite is working as the photoactive layer in the device. Au has been used as the metal contact and was deposited by sputtering. The detailed characteristics of the TiO_2_ compact and mesoporous layer is shown in Fig. S3.†

**Fig. 6:**
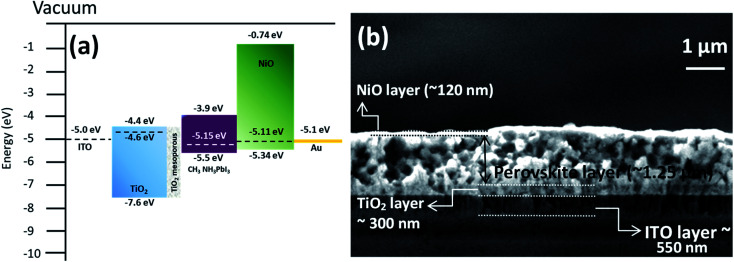
(a) Energy band alignment of the structure consisting of glass/ITO/TiO_2_ compact layer/TiO_2_ mesoporous layer/CH_3_NH_3_PbI_3_/NiO NCs layer/Au. (b) Cross sectional image of the device (glass/ITO/TiO_2_ compact layer/TiO_2_ mesoporous layer/CH_3_NH_3_PbI_3_/NiO) without the metal contact (Au) showing the thicknesses of each layer.

The energy band diagram and band alignment for our device structure are shown in Fig. 6a. The values for the different layers in the band diagram were obtained by measuring the corresponding values of the Fermi level, valence band maximum and bandgap by using a Kelvin probe, XPS and optical absorption spectroscopy.

The cross-sectional view of the device layers ITO/TiO_2_/perovskite/NiO is shown in Fig. 6b, in which each layer is visible distinctly. Fig. S5b† shows an SEM image of the perovskite film top surface, which reveals that the film consists of large grains. The perovskite layer was deposited using spray coating as this approach is perceived to be more suitable for large area coating at a lower cost compared to other thin film deposition techniques.^70,71^ However, spray deposition is a technique not fully developed for device fabrication compared to other methods that have found wide application in research laboratories (*e.g.* spin coating). Spray coating is known to give rise to surface roughness and some other issues are not fully resolved. For instance, spray deposition of perovskite films with an optimized thickness (<500 nm) and suitably large grains remains a challenge.^72^ Here, we have selected the spray deposition conditions in such a way that the degradation of the perovskite crystal structure can be minimized; however a relatively thick perovskite film is produced. Specifically, the TiO_2_ bottom layer provides suitable nucleation sites for the perovskite crystal growth with large grains (Fig. S5b†). The SEM image of the top surface of the NiO NC layer deposited on the perovskite layer is shown in Fig. S5c† and indicates the complete coverage with NiO NCs and a smooth surface.

It has been observed that the device performance is highly dependent on the thickness of the NiO NC layer. The device efficiency with varying NiO NC film thickness is summarized in Fig. S6.† It is noted from Fig. S6† that the efficiency of the devices decreased with increased NiO NC layer thickness. Below 100 nm thickness, the NiO NC layer was not continuous and a patchy coverage was observed from SEM analysis (not shown here). As a result the devices show current leakage and low shunt resistance, which resulted in a low *V*_oc_ and fill factor (FF). When the NiO NC layer is too thick (above 200 nm), the series resistance will increase resulting in a lower fill factor (FF) and a lower short-circuit current density (*J*_sc_). In this work, the best device performance was observed for the thickness range of 100–130 nm of the NiO NC film.

**Fig. 7:**
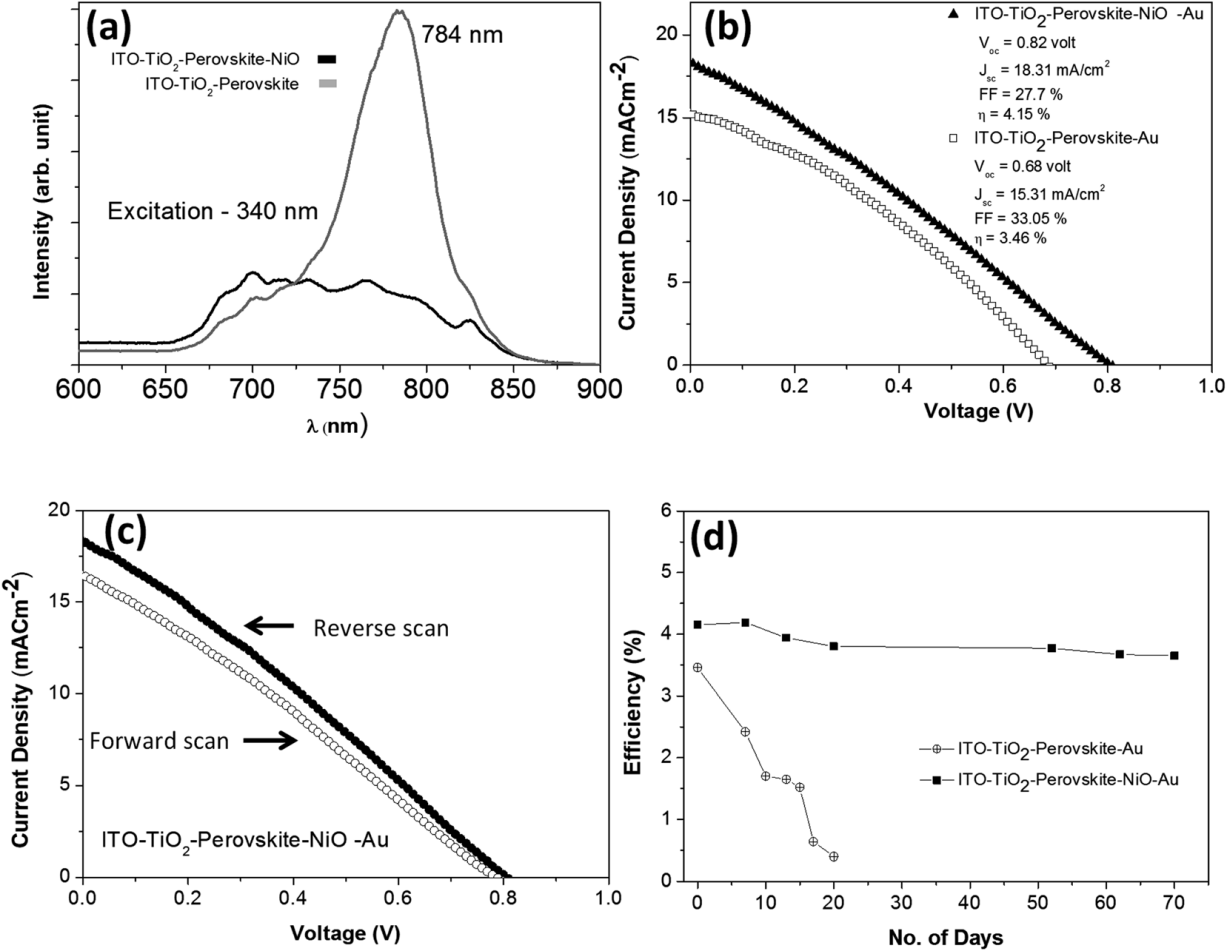
(a) Photoluminescence spectra of CH_3_NH_3_PbI_3_ with and without the NiO NC hole transport layer. (b) Current density *vs.* voltage plot of the metal oxide-perovskite device with and without the NiO NC hole transport layer under one sun conditions (AM 1.5G). (c) Current density *vs.* voltage plot of the metal oxide-perovskite device with a NiO hole transport layer obtained in forward and reverse scans with a delay time of a few seconds and (d) device efficiency stability study over time under ambient conditions of metal oxide-perovskite devices with and without the NiO hole transport layer.

A photoluminescence study was carried out to understand the photo-generated carrier (hole) transport efficiency of the NiO NC film from the perovskite absorber. The photoluminescence spectra of the device with and without the NiO NP layer are shown in Fig. 7a. A significant luminescence quenching can be seen in the presence of the NiO NCs, which suggests efficient carrier dissociation at the interface between the absorber and the transport layer.^73,74^

To further understand the effect of the NiO NC layer, we compared the perovskite device performance with and without the NiO hole transport layer. It was found that the NiO NC film has a significant role in increasing the power conversion efficiency (PCE) of the perovskite device. Around 20% increase in PCE was observed when the NiO NC film was used as an HTL. Fig. 7b shows the comparison of the *JV* plots for the perovskite devices with and without the NiO NC layer. The performance distribution of several devices (ITO/TiO_2_/CH_3_NH_3_PbI_3_/NiO/Au) is shown in Fig. S7.† The *JV* measurements in forward and reverse directions for the metal oxide-perovskite device are shown in Fig. 7c, indicating hysteresis which is normally observed for this type of device.^75,76^ Many reasons have been proposed for such hysteresis in perovskites, like large diffusion capacitance under forward and reverse bias.^77^ The origin of this hysteresis in perovskites is a contentious issue; however a number of probable causes have been proposed. Among them the most supported cause is the presence of mobile ions under an applied electric field; positive and negative ions will migrate towards electrodes and create a temporary dipole. This temporary dipole formation is believed to be the main reason for hysteresis in perovskites.^78–80^ In the case of our solar cell devices, there is no ionic interaction between the perovskite and the metal oxide and hence the ions are free to move with the polarity of the bias voltage, and thus a hysteresis was observed. However, there are several reports which demonstrated NiO_*x*_ hole transport layers for relatively high performance rigid^26,27^ and flexible^23,28^ perovskite solar cells without any clear hysteresis.

The stability of perovskite devices is one of the major issues that will need to be resolved before any commercialization can be achieved. Digital photographs of the perovskite devices freshly prepared and after aging in an ambient atmosphere are shown in Fig. S8(a–d).† Perovskite devices with the NiO NC layer showed stability and no visual degradation (Fig. S8a and b†) was observed even after 70 days of exposure to an ambient atmosphere. However, without the NiO NC top layer, the perovskite started degrading due to moisture contamination from the ambient atmosphere and it is clearly visible in Fig. S8d.† Our results clearly showed that the NiO NC top layer was found to have a two-fold contribution to the improvement of the device performance. First, the NiO NC layer worked as a good HTL and increased the PCE by about 20% compared to the devices without the NiO NC layer. Secondly, the NiO NCs acted as an excellent protective layer for the perovskite, due to their hydrophobicity.^81^ Perovskite devices with and without the NiO NC layer were tested without any encapsulation for around 70 days in an ambient environment at 20 °C and with 20–40% humidity. It was observed that the devices without the NiO NC layer degraded fast and significantly over time (in around 20 days) whereas the devices with the NiO NC protective layer showed good stability during the course of our measurement time (70 days). Fig. 7d shows the efficiency retention performance with time (70 days) for the devices with and without the NiO NC layer. The time evolution of the other device performance parameters (*V*_oc_, *J*_sc_ and FF) and *JV* plots are summarized in Fig. S9(a–c) and S10(a and b).† These results clearly indicate that NiO NCs worked as a protective layer for the perovskite absorber which increased the stability of the solar cell device for a long time. With the NiO NC HTL, the device showed high stability in *V*_oc_, *J*_sc_ and FF (Fig. S9(a–c))† even after exposure to an ambient atmosphere for 70 days, whereas the device without the NiO NC layer showed a significant reduction in *V*_oc_, *J*_sc_ and FF (Fig. S9(a–c))† due to exposure to ambient moisture even for 20 days and finally became inoperative.

## 3. Conclusion

We have successfully demonstrated the efficient synthesis of NiO NCs using atmospheric pressure hybrid plasma-liquid synthesis. A continuous thin film of NiO NCs was deposited by spray coating of the sol. The materials and energetic properties of the NiO NCs have shown ideal characteristics for their implementation in solar cell devices. As a proof of concept, we have demonstrated the general applicability of NiO NCs as a promising HTL. Quantum confined NiO NCs showed excellent band alignment for Si-QD, N-CQD and perovskite absorber based solar cells. The NiO NC HTL significantly improved the stability of perovskite solar cells and improved the carrier transport for the perovskite. The perovskite-based device with the NiO NC HTL showed excellent environmental stability even after 70 days of exposure to ambient moisture. Our results indicate that atmospheric pressure hybrid plasma-liquid synthesis of NiO NCs offers great opportunities for the integration of this technique into device fabrication steps. Also, from these results, it is clear that NiO NCs and other quantum confined metal oxides represent a powerful set of materials that could facilitate band alignment in a wide range of next generation device architectures as well as offering opportunities to prevent device degradation. Further modification of the NiO HTL by incorporating other materials for improving the film quality, electrical conductivity and hole extraction ability can be interesting research to carry out.

## Conflicts of interest

There are no conflicts to declare.

## Supplementary Material

NA-001-C9NA00299E-s001
