# Supplementary material for: Microplasma-synthesized ultra-small NiO nanocrystals, a ubiquitous hole transport material
Source: Nanoscale Adv. 2019 Oct 22;1(12):4915–25. doi: 10.1039/c9na00299e (PMC9417055; doi:10.1039/c9na00299e)
Supplement: NA-001-C9NA00299E-s001 [file NA-001-C9NA00299E-s001.pdf]

Electronic Supplementary Material (ESI) for Nanoscale Advances.
This journal is © The Royal Society of Chemistry 2019

Supporting Information

Microplasma-synthesized ultra-small NiO nanocrystals, an ubiquitous hole transport material for next generation solar cells

Supriya Chakrabarti*[ab], Darragh Carolan[a], Bruno Alessi[a], Paul Maguire[a], Vladimir Svrcek[c] and Davide Mariotti[a]

[a]Nanotechnology & Integrated Bio-Engineering Centre (NIBEC), Ulster University, Jordanstown, Newtownabbey, Co. Antrim, BT37 0QB, UK. E-mail: supriya.c@arci.res.in

[b]Centre for Carbon Materials, International Advanced Research Centre for Powder Metallurgy and New Materials (ARCI), Balapur P.O., Hyderabad, 500005, India

[c]National Institute of Advanced Industrial Science and Technology (AIST), Department of Energy and Environment, Research Center of Photovoltaics, Advanced Processing Team, Central 2, Umezono 1-1-1, Tsukuba, Ibaraki, 305-8568, JAPAN

*Corresponding authors: *Email addresses*: supriya.c@arci.res.in / supriyac79@gmail.com (Supriya Chakrabarti)

1. Experimental

1.1 Synthesis of NiO nanocrystals

Atmospheric pressure direct current (DC) microplasma interacting with the liquid phase [1, 2] has been employed to synthesize NiO NCs in ethanol. Supplementary Fig. S1a shows the schematic diagram of the hybrid plasma-liquid setup for synthesizing NiO NCs. 99.5% purity grade Ni foil of thickness 0.1 mm purchased from Good Fellow ltd. was used as a source of Ni and as anode by immersing in 15 mL of ethanol (immersed area of the Ni foil was maintained to be 1.5 cm x 1.5 cm for each experiment). A Ni tube (0.7 mm internal diameter and 1 mm external diameter) was used as cathode for this synthesis. The distance between anode and cathode was kept constant at about 1.8 cm for all the synthesis in this report. Pure He gas flow at a flow rate of 50 standard cubic cm (sccm) through the Ni tube and initial voltage of 3 kV were used to create the plasma between the end of the nickel tubing and the surface of ethanol. The distance between the Ni tube and the surface of the liquid was kept at 2 mm throughout the experiment. The reaction was paused and the solution stirred every 10 min.

At the starting point a DC voltage of 3 kV was set and applied until the current reached 5 mA. As soon as the current reached the 5 mA, the current was maintained constant by gradually decreasing the voltage from 3 kV to 2 kV. A total processing time of 45 min was used for each synthesis of NiO NCs. The formation of NiO NCs started as soon as the microplasma was generated. The concentration of NiO NCs in ethanol can be varied by changing the processing time and current. Supplementary Fig. S1b shows the digital

photographic image of the hybrid plasma liquid reactor with He plasma in action to synthesize NiO NCs.

1.2 Solar cell fabrication

1.2.1 TiO_2 electron transport layer

The TiO_2 compact layer was deposited onto indium-doped tin oxide (ITO) coated glass substrate using a sol-gel technique [2]. The sol of TiO_2 was prepared by mixing Titanium(IV) isopropoxide (1.56 mL), ethanol (18 mL) and triethanolamine (0.39 g) under constant stirring on hot plate at 40°C for 2 h. The solution was kept overnight and then was spin coated on the substrate at 5000 revolution per minute (rpm) for 30 s followed by annealing at 350°C for 4 h.

For making the TiO_2 mesoporous layer, TiO_2 Dyesol paste (18NR-T) and ethanol was mixed in the weight ratio of 1:4 and followed by ultra-sonication for 2 h. The ultra-sonication helped to get stable well dispersed anatase TiO_2 NCs solution suitable for thin film deposition. The solution was then spin coated onto the TiO_2 compact layer coated ITO glass substrate at 2000 rpm for 60 s. After the spin coating the substrate was heated at 100°C for 10 min and then annealed at 400°C for 2 h to get a transparent sintered TiO_2 mesoporous layer.

Fig. S3a shows the surface morphology of the TiO_2 layer obtained by spin coating. The TiO_2 layer shows a smooth and continuous surface suitable for perovskite layer growth. Fig. S3c shows the cross sectional SEM image of TiO_2 compact layer and TiO_2

mesoporous layer onto the ITO coated glass substrate. The image reveals that the thickness of TiO_2 compact layer and TiO_2 mesoporous layer is about 300 nm altogether. The XPS spectrum of TiO_2 is shown in Fig. S3b which reveals two distinct peaks at 458.4 eV for the Ti $2p_{1/2}$ and at 464.2 eV for the Ti $2p_{3/2}$, both in good agreement with the literature [3]. The TiO_2 layer shows good optical transmittance (Fig. S3d).

1.2.2 Perovskite absorber layer

Methylammonium lead iodide ($CH_3NH_3PbI_3$) perovskite powder with 99% purity (purchased from Xi'an p-OLED, China) was mixed in dimethylformamide (DMF) under stirring at 60 °C at 0.125 M (for $CH_3NH_3PbI_3$ 1 M = 0.619.9 g/mL). The solution was then spray coated using an airbrush (Pro BD-132) with nitrogen gas flow at 1 bar in air onto the mesoporous TiO_2 layer and annealed for 40 min at 100 °C.

1.2.3 NiO nanocrystals hole transport layer

A continuous layer of NiO NCs was deposited onto the perovskite layer using an airbrush (Pro BD-132) spray coater. 1 mL of NiO-ethanol sol via nitrogen gas flow at 1 bar was spray coated onto the perovskite layer followed by annealing for 10 min at 120°C. During the spray coating of NiO-ethanol sol no degradation of the perovskite layer was observed as the contact time of ethanol with perovskite layer was negligible, before ethanol can damage the perovskite layer it evaporated at 120°C.

1.2.4 Gold (Au) metal contacts

Au metal contact was deposited onto the NiO NCs hole transport layer using a sputtering technique (Moorfield minilab DC/RF magnetron box sputter system). Argon plasma at constant current of 0.15 A for 60 min with a working pressure of 1.5×10^{-2} mbar was used to sputter Au from a 99.99% pure Au target.

1.2.5 Silicon quantum dots layer

Thin films of Si-QDs have been deposited using a flat parallel electrode atmospheric-pressure plasma system where silane (SiH_4) was used as precursor, being delivered directly into the reactor together with the other gases. The details of the synthesis process and material characterization have been reported elsewhere [4-6].

1.2.6 Nitrogen doped Carbon Quantum dots layer

N-CQDs were synthesized using atmospheric pressure microplasma-liquid interactions. The atmospheric pressure microplasma setup consists of a direct-current (DC) source, a carbon rod as the cathode and a hollow nickel tube as the anode. At first citric acid (1.051 g, 7.8 mmol of COOH groups) and ethylenediamine (556 µL, 15.6 mmol NH_2 groups) were mixed in 10 mL de-ionized (DI) water (1:2 molar ratio of COOH:NH_2) to make a solution suitable for the synthesis of N-CQDs. The carbon electrode was immersed in this solution while the nickel tube was brought close to the surface of the liquid and the gap was maintained within 2 mm throughout the experiment. The distance between anode and cathode was approximately 2 cm. Pure He gas flows at 60 sccm through the Ni tube and a current of 6 mA between the electrodes were used to create

the microplasma. The distance between the nickel tubing and the surface of the water was adjusted to maintain a constant discharge voltage of 1.3 kV. The reactions were carried out for 30 minutes in total to give a yield of 1 mg/mL of N-CQDs in water. The detail synthesis process and material characterization have been reported in a separate communication [7].

2. Characterization

2.1. Materials characterization

Transmission electron microscopy (TEM) was carried out using a JEOL JEM-2100F equipped with a field emission electron gun operated at 200 kV. Atmospheric plasma synthesized NiO nanocrystals-ethanol colloids with no further purification was directly dropped on a holy carbon coated Cu grid of 300 meshes and evaporated overnight to prepare the samples for TEM study.

The chemical compositional analysis was performed by X-ray photoelectron spectroscopy (XPS) using an X-ray source (Al = 1486 eV) and the Kratos Axis Ultra DLD spectrometer. X-ray spot size was 400 μm^2. The sample analysis chamber pressure was maintained at 10^{-9} bar for all measurements. Current and voltage were 10 mA and 15 kV respectively during the measurements. The NiO nanocrystals in ethanol without further purification was spray coated onto Si (N-type, 100) substrate for XPS analysis. Specific region scans were performed at a resolution of 0.05 eV and pass energy of 20 eV. Calibration was performed using the C 1s peak located at 284.5 eV.

The photoluminescence measurement was done using an Agilent Cary Eclipse Fluorescence Spectrophotometer equipped with high voltage Xe flash lamp operating at 1260 kPa. The photovoltaic devices with (glass/ITO/TiO_2/Perovskite/NiO) and without NiO layer (glass/ITO/TiO_2/Perovskite) were directly placed with the help of solid sample holder for photoluminescence measurements.

Scanning electron microscopy (SEM) images were obtained with a JEOL JSM-6010 PLUS operating at 20 kV acceleration voltage. The materials were spray coated on ITO coated glass substrates for SEM analysis.

A scanning Kelvin probe system from KP-technology was used to estimate the work function of the samples. The samples were prepared by spray coating of materials onto ITO-coated glass substrates.

Ultraviolet-visible (UV-Vis) spectra of the samples were measured by Perkin Elmer Lambda 650S using a deuterium-halogen light source and an integrating sphere. Directly synthesized colloidal solutions of nanocrystals were transferred into quartz substrates for UV-VIS spectroscopy.

X-ray di□raction (XRD) measurements were performed with a Bruker di□ractometer with a Cu K_α line of 0.1541 nm.

2.2 Device characterization

Current density–voltage (JV) curves of solar cell devices were recorded using a Keithley 6430 sub-FA source meter unit under illumination (1.5 AM) in air at ambient

temperature. The applied bias varied from -1 V to 1 V in steps of 0.01 V at a scan rate 700 mV/s for all the measurements.

References:

1. G. F. Brown and J. Wu, Laser Photon. Rev., 2009, 3, 394-405.

2. C. Rocks, V. Svrcek, P. Maguirea and D. Mariotti, J. Mater. Chem. C, 2017, 5, 902-916.

3. C.D. Wagner, A.V. Naumkin, and A. Kraut-Vass, NIST X-ray Photoelectron Spectroscopy Database 20, Version 3.5 2003.

4. M. Macias-Montero, S. Askari, S. Mitra, C. Rocks, C. Ni, V. Svrcek, P. A. Connor, P. Maguire, J. T. S. Irvine and D. Mariotti, Nanoscale, 2016, 8, 6623–6628.

5. S. Askari, M. Macias-Montero, T. Velusamy, P. Maguire, V. Svrcek and D. Mariotti, J. Phys. D: Appl. Phys., 2015, 48, 314002-314020

6. S. Askari, I. Levchenko, K. Ostrikov, P. Maguire, and D. Mariotti, Appl. Phys. Lett., 2014, 104, 163103-5

7. D. Carolan, C. Rocks, D. Padmanaban, P. Maguire, V. Svrcek and D. Mariotti, Sustainable Energy and Fuels, 2017,1, 1611-1619

Supporting Figures

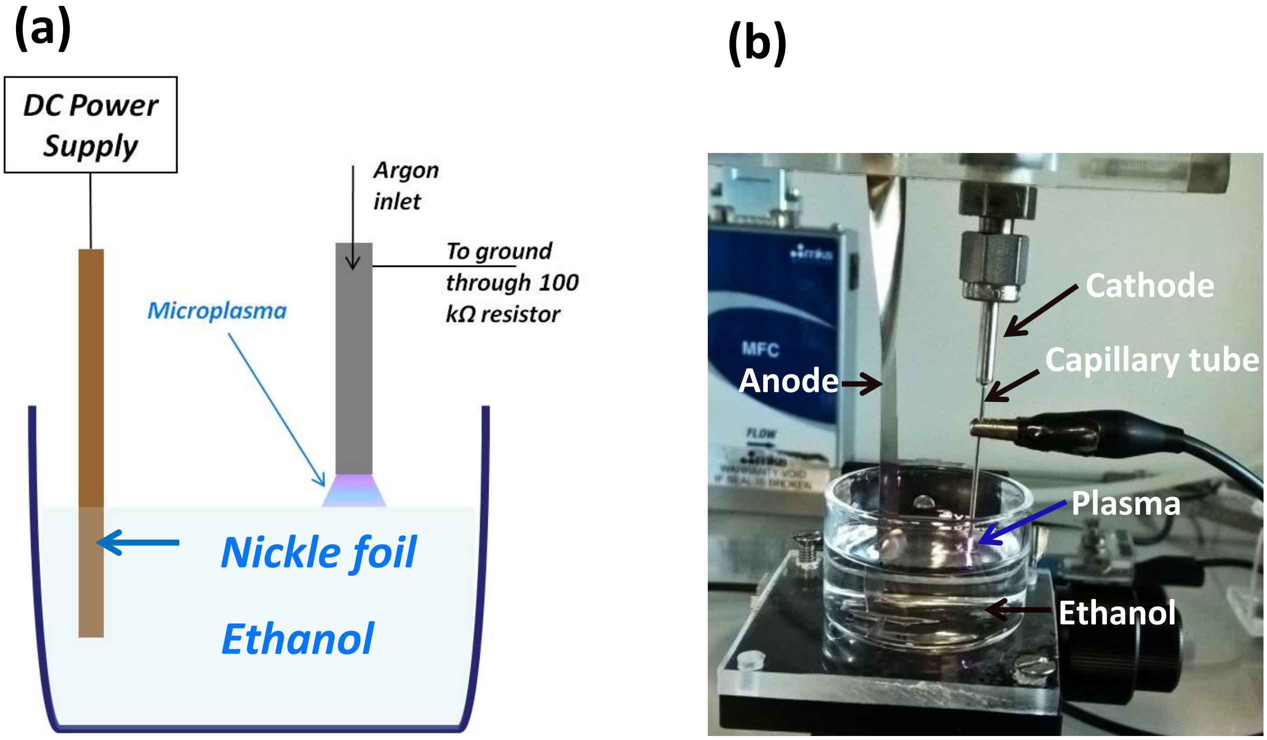

Figure S1 The atmospheric pressure hybrid plasma liquid synthesis unit: (a) schematic diagram of the system and (b) digital photographic image of the system showing all components.

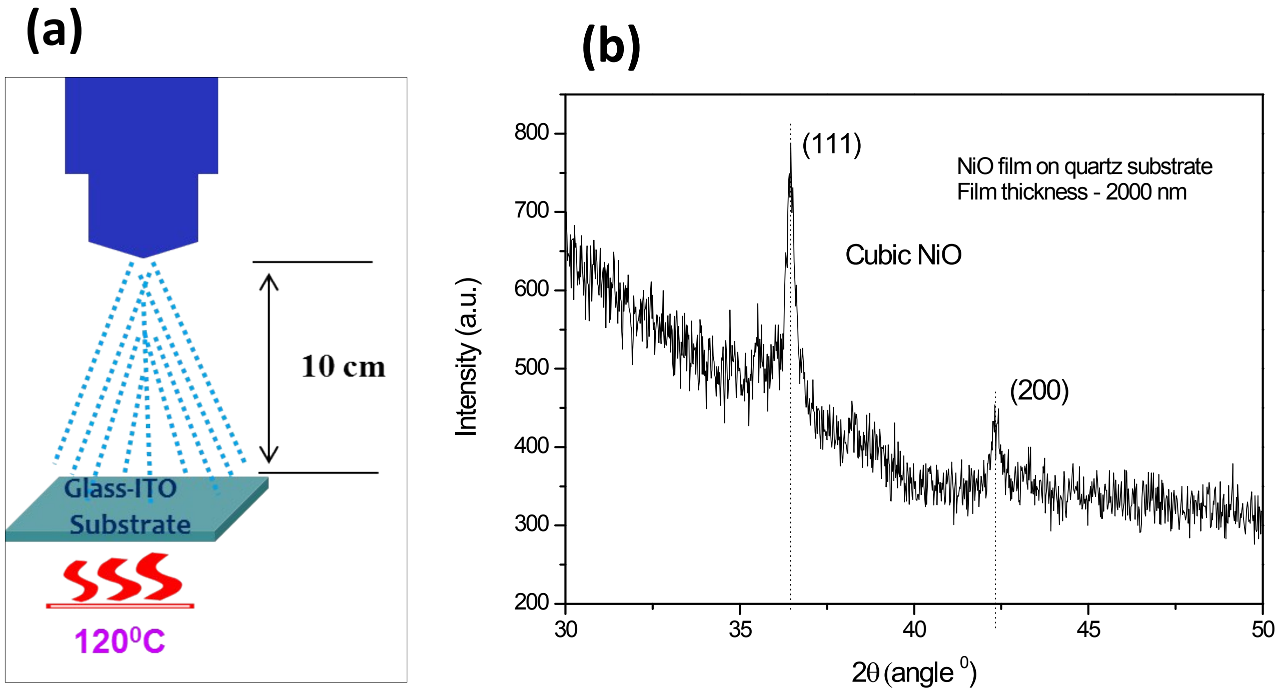

Figure S2 (a) Schematic diagram of spray deposition systems for NiO NCs layer deposition from NiO-ethanol colloidal sol, (b) XRD spectrum of NiO NCs film spray deposited on quartz substrate and the thickness of the film is ~ 2000 nm.

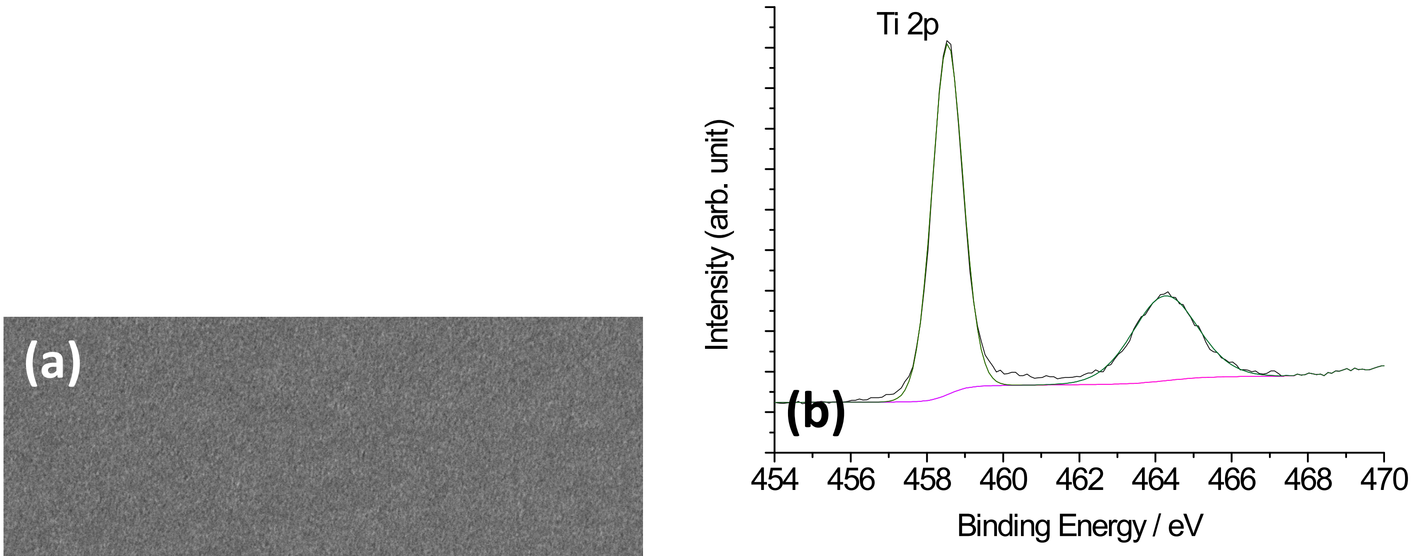

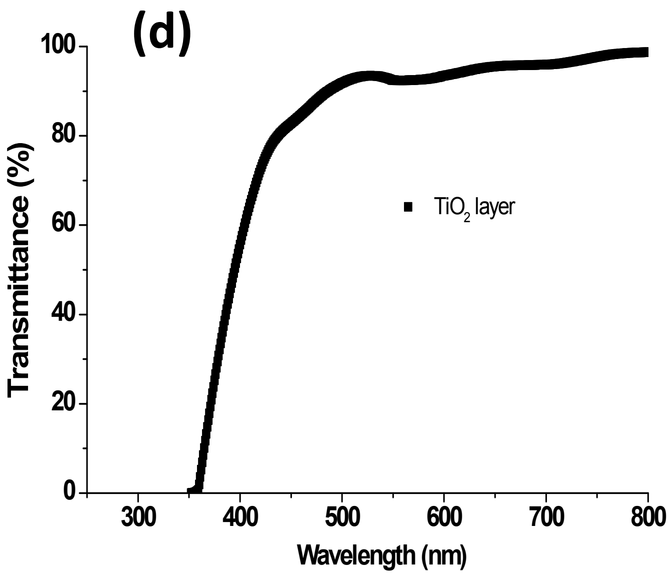

Figure S3 (a) SEM image of the top surface of TiO_2 compact layer/TiO_2 mesopurous layer deposited onto substrate. (b) Cross sectional SEM image of the TiO_2 compact layer/ TiO_2 mesopurous layer showing two different layers distinctly. (c) XPS spectrum of Ti 2p peaks. (d) Optical transmittance spectra TiO_2 compact layer/TiO_2 mesoporous layer (~300 nm) films on quartz substrate.

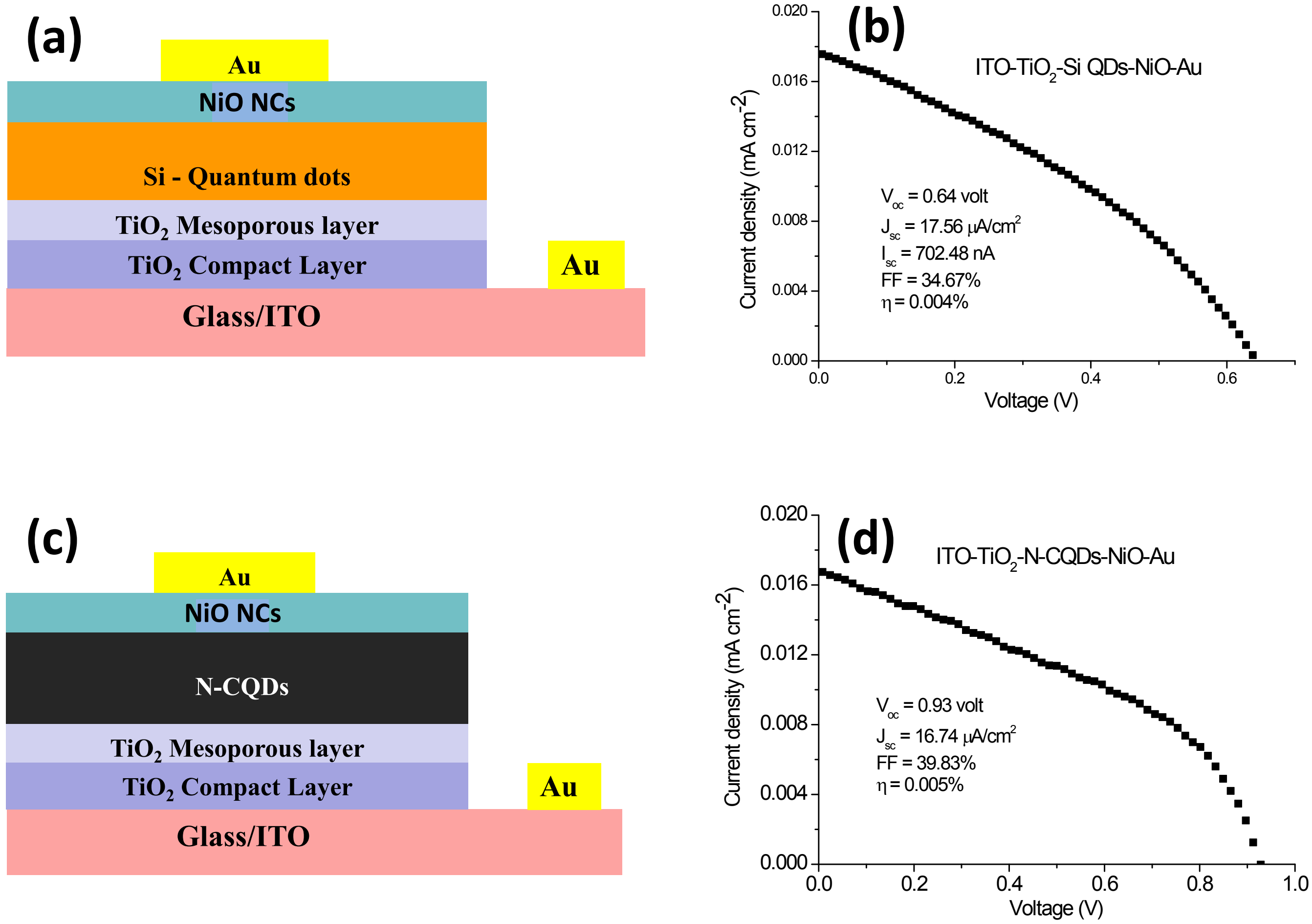

Figure S4 (a) Complete all-inorganic Si-QDs based device structure consisting of glass/ITO/TiO_2 compact layer/TiO_2 mesoporous layer/Si-QDs/ NiO /Au. (b) current density vs. voltage plot of the all-inorganic Si-QDs based device with NiO NCs hole transport layer under one sun condition (AM 1.5G). (c) Complete all-inorganic N-CQDs based device structure consisting of glass/ITO/TiO_2 compact layer/TiO_2 mesoporous layer/N-CQDs/ NiO/Au. (d) Current density vs. voltage plot of the all-inorganic N-CQDs based device with NiO hole transport layer under one sun condition (AM 1.5G).

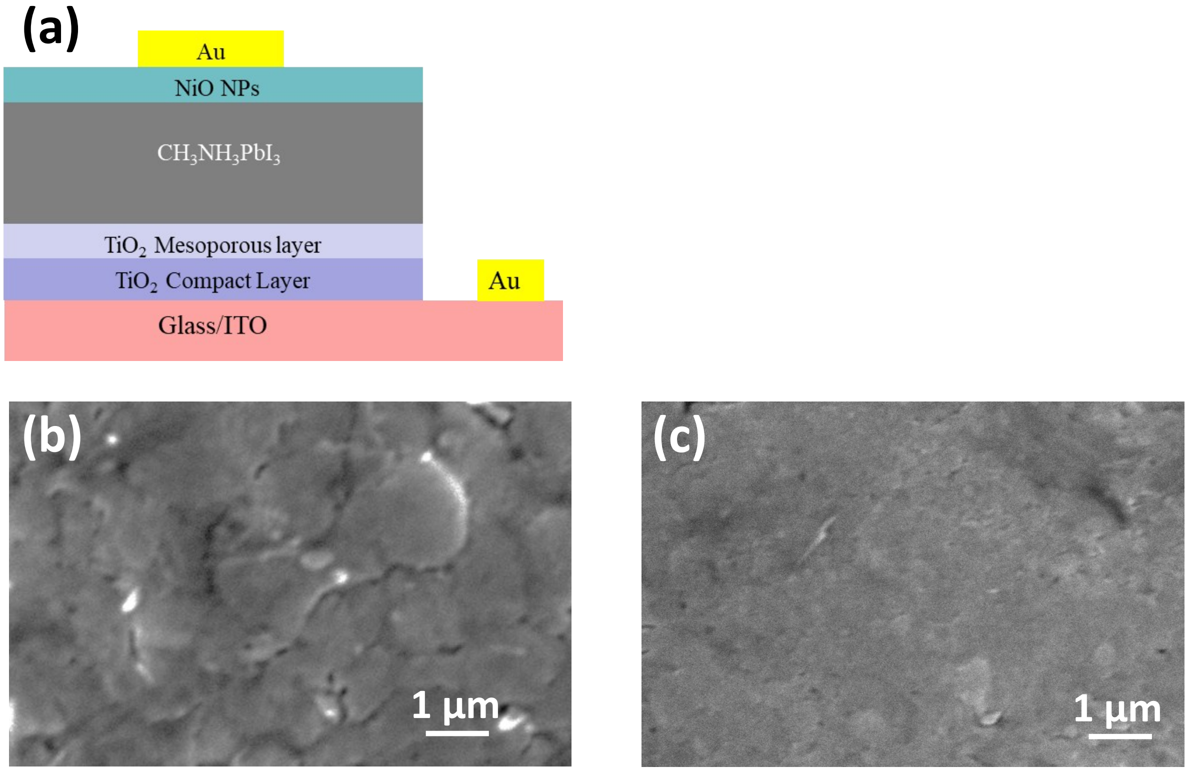

Figure S5 (a) Complete device structure consisting of glass/ITO/TiO_2 compact layer/TiO_2 mesoporous layer/$CH_3NH_3PbI_3$/NiO NCs layer/Au, (b) SEM image of top surface of Perovskite layer grown on TiO_2 layer and (c) SEM image of top surface of Perovskite layer coated with NiO NCs layer.

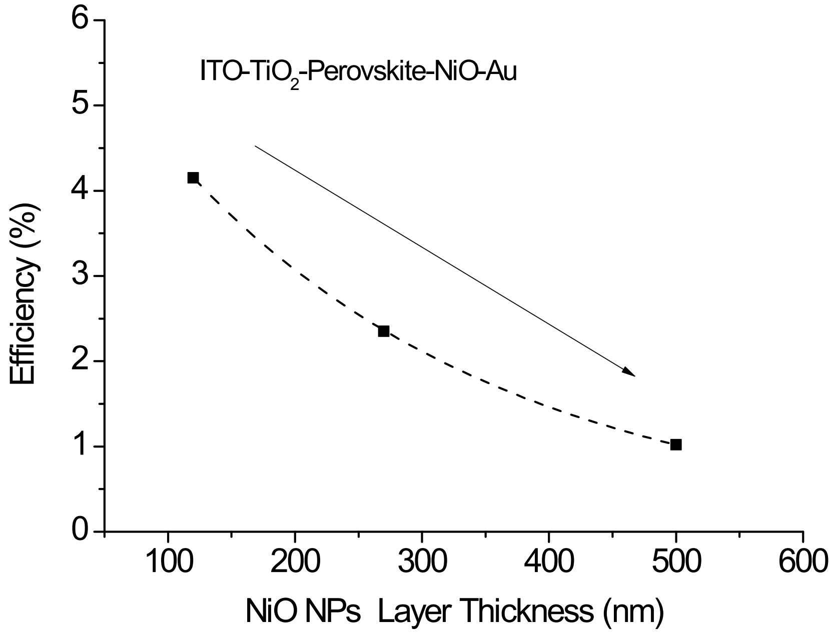

Figure S6 NiO NCs layer thickness dependence of the glass/ITO/TiO_2 compact layer/TiO_2 mesoporous layer/$CH_3NH_3PbI_3$/NiO/Au device efficiency under one sun condition (AM 1.5G).

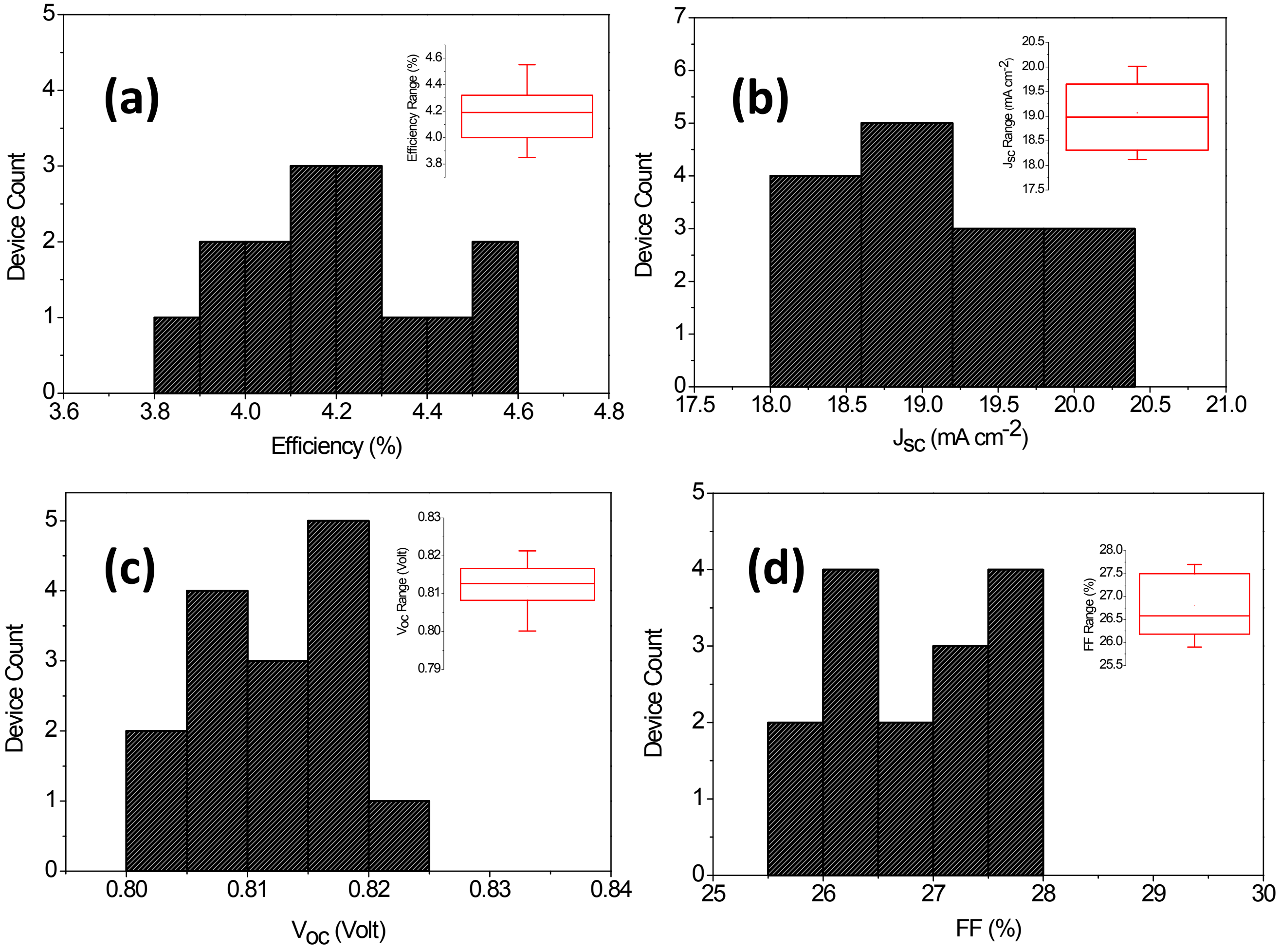

Figure S7 Performance distribution of several devices (ITO/TiO_2/$CH_3NH_3PbI_3$/NiO/Au): (a) efficiency, (b) J_{SC}, (c) V_{OC} and (d) FF. The corresponding insets show the statistical box charts of performance distribution.

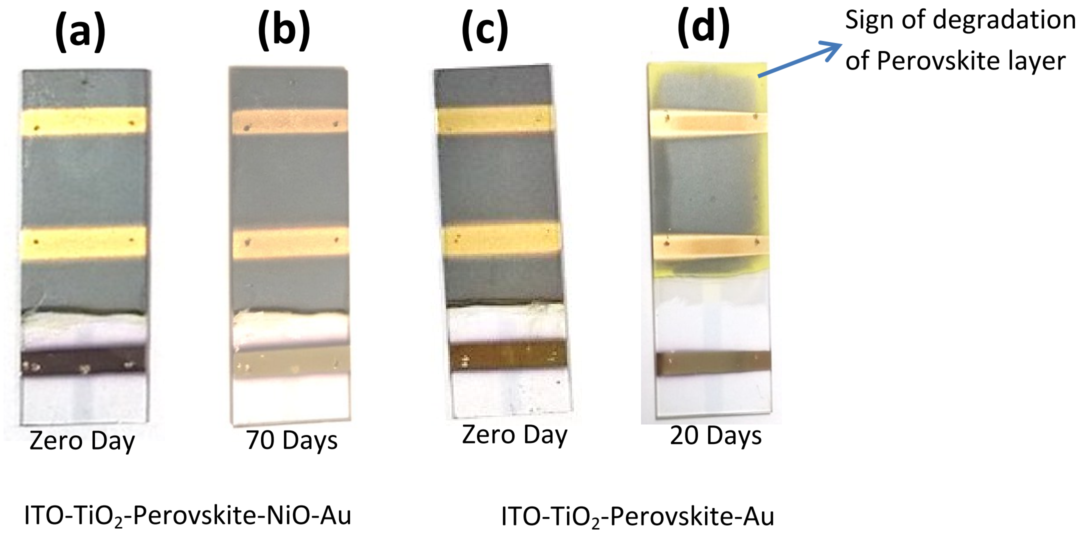

Figure S8 Digital photographic image of (a) freshly prepared metal oxide perovskite device with NiO hole transport layer, (b) same device after 70 days of aging in ambient atmosphere, (c) freshly prepared metal oxide perovskite device without NiO hole transport layer and (d) device after 20 days of aging in ambient condition showing degradation of perovskite layer (the transformation from black to yellow colour indicates the oxidation of perovskite layer).

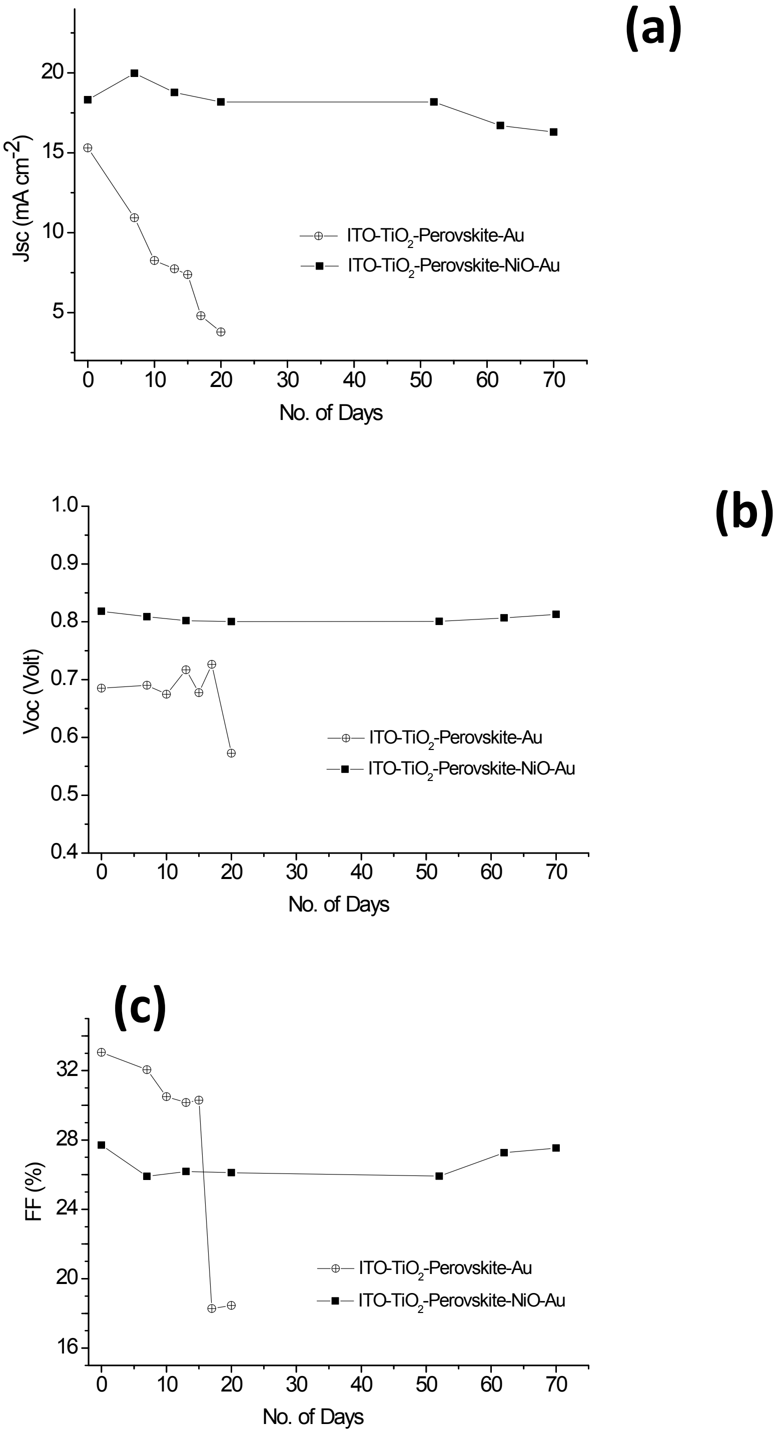

(a)
Jsc (mA cm-2)
20
15
10
5
0
10
20
30
40
50
60
70
No. of Days
ITO-TiO2-Perovskite-Au
ITO-TiO2-Perovskite-NiO-Au
(b)
Voc (Volt)
1.0
0.9
0.8
0.7
0.6
0.5
0.4
0
10
20
30
40
50
60
70
No. of Days
ITO-TiO2-Perovskite-Au
ITO-TiO2-Perovskite-NiO-Au
(c)
FF (%)
32
28
24
20
16
0
10
20
30
40
50
60
70
No. of Days
ITO-TiO2-Perovskite-Au
ITO-TiO2-Perovskite-NiO-Au

Figure S9 Device stability study over time in ambient condition of metal oxide-perovskite device with and without NiO hole transport layer: (a) short-circuit current density, (b) open-circuit voltage and (c) fill factor.

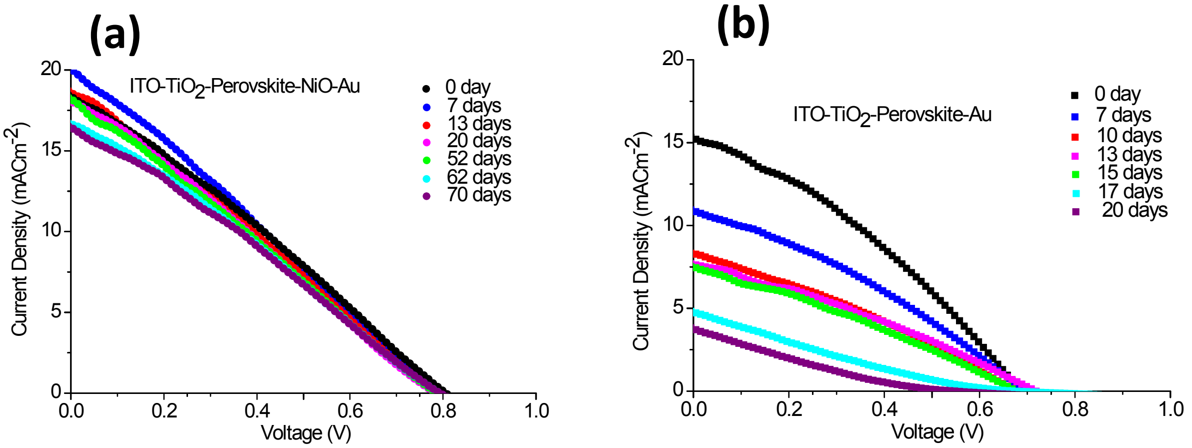

Figure S10

Current density vs. voltage plot of the metal oxide-perovskite device under one sun condition (AM 1.5G) for different number of days exposed in ambient atmosphere (a) device with NiO hole transport layer and (b) device without NiO hole transport layer.
